# The Medical Aspects of Dried Milk

**Published:** 1921-10

**Authors:** 


### MEDICAL ASPECTS OF DRIED MILK.

(By a Correspondent.)

The rapid extension of the use of dried milk seems one of the romances of modern dietetics. Previous to 1908 many methods of drying milk had been attempted, but the bugbear had been to produce a whole cream milk powder, which came up to Board of Trade standard as regards its cream content, and which did not become rancid on keeping. Modern methods of machinery have overcome this difficulty.

### Manufacture.

There are two main methods of desiccating milk. 1. The spray process, by which milk condensed to a certain degree *in vacuo* is pumped through a fine nozzle into a chamber through which hot air circulates. 2. The hot-roller process, of which there are many modifications, but in which the principle is the rapid evaporation of the water by passing the milk over rapidly revolving heated cylinders. In New Zealand, whence the bulk of full cream dried milk used in this country comes, modifications of the hot-roller process are used, but precautions are taken to ensure that little time is lost from the milking of the herds to the desiccation of the liquid.

It may be well to review briefly the changes which occur in milk during desiccation, for it is by an understanding of these changes that many of its clinical advantages can be explained. The sugar content is unchanged. The globules of fat seem to undergo some physical change, with the result that in the reconstituted milk they tend to come to the surface, and have a slightly more yellowish tinge, resembling the fat of butter. Sommerville considers that since the fat is present for the most part of fatty acids, it is more readily absorbed in the small intestine than the fat of ordinary cows' milk. The protein seems to undergo some molecular change, for all observers agree that it is more easily digested than the caseinogen of liquid milk, and in the reconstituted milk the clot formed on the addition of rennin no longer resembles the heavy casein clot, but is light and flocculent.

What, however, is of greater importance than any chemical or physical alteration that desiccation may bring about, is the fate of the all-important accessory food factors. Fat Soluble "A"—the so-called anti-rachitic factor—richly present in butter fat, is entirely unchanged; the second accessory food factor "B"—anti-neuritic—is present in milk in large amounts, and entirely withstands drying. The third factor, water soluble "C"—anti-scorbutic—was originally thought to be slightly diminished in potency during the process of desiccation, but later work by Hess in America, and other investigators in this country show that the diminution is practically nil when the milk, drawn from herds fed on green pasture is dried by the rapid hot-roller process. It has been demonstrated that cows fed on fresh green food give a milk richer in vitamins than those that are stall-fed; it is essential that there should be no alkali added, and that little time should elapse from milking to desiccation.

Perhaps the most striking feature of dried milk is its great bacterial purity; desiccation practically sterilises the milk. Whereas in the so-called "nursery" milks up to 20,000,000 germs per c.c. are found, and in pasteurised milk as many as 80,000—many of these pathogenic in type—in a good full cream dried milk, reconstituted, 200 organisms per c.c. is the rule, and these harmless saphrophytes. The different brands of dried milks vary somewhat in their bacterial purity, depending on the care in the handling of the original milk and the subsequent packing. One remarkable feature, and important in these days of tuberculous herds, is the elimination of the tubercle bacillus. Professor Delépine has shown that during the process of drying even milk purposely infected loses its virulence. Fortunately most of the dried milk used in Great Britain, in any case, comes from carefully tested cows in the Antipodes, where open grazing in a temperate climate makes bovine tuberculosis rare.

A brief survey, then, shows us that freshly-drawn milk, dried by the rapid hot-roller process, is bacterially very pure, and that there is practically no change in its chemical constitution beyond rendering it more assimilable, and also, granted the source is correct, and the handling of the original milk not delayed, its vitamin potency is unimpaired.

### As an Infants' Food.

Unfortunately during the last few decades there has been a general falling off of breast-feeding; social and economic conditions having tended to this, and, although much good work to encourage normal feeding is being done by Welfare Centres, it is hoped that the wider extension of ante-natal clinics will still further this.

Cow's milk, easy to obtain, and formerly moderate in price, has been the alternative of choice, but modern milk is a mysterious mixture in these days of insufficient veterinary and sanitary supervision, delayed transport, and careless handling. Under the present conditions the problem of supplying at a moderate price a wholesome, clean milk seems well-nigh impossible.

Recent analysis has shown, except in the case of specially treated grade "A" milk, a decline in purity of the urban milk supplies. Bovine tuberculosis is on the increase, whilst even bulk pasteurisation as practised on practically all the milk that comes to London has little effect in keeping down contamination by B.C.C. and streptococci, and even more virulent organisms.

Granted that our milk supply was ideal, from a bacterial point of view, cows' milk, with its high protein content, without being diluted and cream and sugar added, is not the ideal mixture for an infant. So modifying milk entails expense, knowledge, and some skill, also much handling of a liquid not already bacterially very clean. Such modification is often beyond the power of a mother

**Medical Aspects of Dried Milk—(*cont.*).**

to provide. For a time whole-milk feeding with citration was the vogue, but here again the mother is severely handicapped by an impure milk supply, whilst recent knowledge has shown that alkalisation plays havoc with the anti-scorbutic factors. Boiled and sterilised milk have the objection that there is a marked destruction of the anti-scorbutic properties.

Artificial foods were utilised for many years. Several of these are quite good as temporary measures, but practically all were deficient in fat, and contained an excess of carbohydrates, in many cases in the form of starch. The profession regarded them askance, always fearing that their mode of manufacture entailed destruction of the vital properties.

The advent, then, of a clean full-cream dried milk has played an important part in modern infant dietetics; its advantages are: its ease of transport, its freedom from waste, its purity, ease of preparation, assimilability, and the minimising of the risk of the deficiency diseases, rickets and scurvy. Most healthy children can take a full-cream dried milk from early infancy, yet in some cases it is necessary either to employ a half-cream mixture, or even a skimmed dried milk with added milk-sugar, until such a time as is established their tolerance for fat. In premature and weakly infants this is always advisable.

### Over-feeding.

The worst criticism that can be levelled at the use of dried milk is the tendency to over-feed. Most manufacturers give their directions on the tin in teaspoonfuls—a most elastic measure, since a teaspoon can contain anything from 55 to 85 grams, according to the shape and how it is filled. It should be incumbent on every manufacturer to supply an exact measure with the tin, and to emphasise the necessity of using this, for there is a great temptation on the mother's part to give a little extra so as to fatten baby. Constipation is commonly seen amongst babies fed on dried milk; and in most cases this will be found to be due to over-feeding. Again, the directions issued by many firms are grossly on the generous side, especially in the earliest weeks and the later months.

### The Sick Child.

Infants suffering from dyspepsia, or atrophy, tolerate and digest dried milk very well, and the fact that it can be given in a concentrated form is useful in cases of vomiting and gastric intolerance. As a prophylactic against "Summer" diarrhœa the results have been uniformally excellent, as would be expected from its purity and unlikelihood of contamination. Also, since it is easily digested, its use precludes that initial gastric disturbance so common in children during hot, dry spells, and which, if neglected, passes on to the more severe gastro-enteritis. In the treatment of infective gastro-enteritis, after the initial spell of boiled water, infants will tolerate a diluted mixture of dried milk, preferably half-cream, much sooner than ordinary cows' milk.

### Mixed Feeding.

In mixed feeding dried milk has been termed "Alimentation de choix"; in cases where the breast-milk is insufficient it can be supplemented by dried milk, the fact that the protein element resembles that of mothers' milk removes the difficulty which was experienced when a cows'-milk mixture is used.

In those mothers who have to go to employment the period of lactation can be maintained by alternate feeding. We are of the opinion that by the exercise of a little instruction and patience much can be done by Centres in educating the working mother how to utilise a dried milk during the working hours, and encourage her to maintain her own milk for night and morning feeds.

In its employment for adults generally advantage can be taken of the possibility of giving a dried milk of any suitable concentration, and it need not be always diluted to the strength of ordinary cows' milk. In this way one can avoid the excessive bulk so often necessary, and in cases of malnutrition, phthisis, and after fevers, it can in this way supply extra nourishment in a palatable form. For invalids generally we think the possibilities of dried milk have not been quite sufficiently explored.

### Use in the Tropics.

Of late much attention has been drawn to the extensive possibilities of dried milk in tropical and sub-tropical countries, where not only is the milk supply poor, but the quality and keeping properties practically impossible. In India three-quarters cream-dried milk has been found the most serviceable, as a child in the tropics seems to have lessened tolerance for fat as compared with the English child. In the treatment of dysentery, sprue, and other intestinal disorders, dried milk has proved a great boon. On board ship, and in countries like Malta, where it is impossible to utilise the local supply, dried milk should prove a great advantage.

---

Sir William Hartley, who previously gave the Jubilee Cottage Hospital to Colne, East Lancashire, has now laid the foundation-stone of the new Hartley Hospital, which he has undertaken to present to the town. It will adjoin the Hartley Homes for the Aged, another of his gifts. The new hospital, which has had the benefit of Dr. Mackintosh's advice in regard to its construction, will accommodate forty patients. Attached to the gift, which includes the equipment of the building, is the condition that it should be maintained adequately, and the donor was able to announce when laying the foundation-stone that the financial position was assured by the weekly contribution scheme which has already been well supported. When the Jubilee Cottage Hospital was founded, the donor made it a condition that an endowment equal to the cost of the institution should be formed. The success of the contribution scheme, in advance of the completion of the building, shows what can be done, and promises better for the future than handsome donations by themselves.

